# Effects of vildagliptin as add-on treatment in patients with type 2 diabetes mellitus: insights from long-term clinical studies in Japan

**DOI:** 10.1186/s40200-016-0240-z

**Published:** 2016-07-04

**Authors:** Masato Odawara, Rieko Sagara

**Affiliations:** 1 The Department of Diabetology, Endocrinology, Metabolism and Rheumatology, Tokyo Medical University, 6-7-1, Nishi-Shinjuku, Shinjuku-ku, Tokyo 160-0023 Japan; 2 Medical Division, Novartis Pharm K.K, Toranomon Hills Mori Tower, 23-1, Toranomon 1-chome, Minato-ku, 105-6333 Tokyo Japan

**Keywords:** Combination therapy, Dipeptidyl peptidase-4 inhibitor, Long-term administration, Oral antidiabetes drugs, Vildagliptin

## Abstract

**Background:**

Vildagliptin, a dipeptidyl peptidase-4 (DPP-4) inhibitor, is wildly used to treat type 2 diabetes mellitus (T2DM) with mono- or combination-therapy. We review two previously published open-label studies to extract insights on the long-term efficacy and safety of vildagliptin.

**Methods:**

Two studies were conducted in Japan to assess the efficacy and safety of vildagliptin as an add-on to other oral antidiabetes drugs (OADs) for 52 weeks. These studies were performed under the similar protocol in Japanese patients with T2DM who were inadequately controlled with OAD monotherapy [excluding other dipeptidyl peptidase-4 (DPP-4) inhibitors].

**Results:**

Addition of vildagliptin (50 mg twice daily) to other OAD monotherapy [sulfonylurea (SU), metformin, thiazolidinedione, alpha-glucosidase inhibitor and glinide] reduced glycated hemoglobin (HbA1c) levels by −0.64 %,−0.75 %,−0.92 %,−0.94 % and − 0.64 %, respectively, over 52 weeks of treatment. Overall, the incidence of hypoglycemia was low and was slightly higher in the add-on to SU treatment group compared with the other groups. The incidences of adverse events were comparable among the treatment groups, and vildagliptin was well-tolerated as add-on therapy to other OADs.

**Conclusions:**

The evidence from the two studies indicates that vildagliptin as an add-on therapy to other OADs is a clinically reasonable option for Japanese patients with T2DM who respond inadequately to other OAD monotherapy.

## Background

The Japanese guideline [1] and the international guidelines [2] for management of patients with type 2 diabetes mellitus (T2DM) recommend maintaining tight glycemic control to suppress aggravation and/or occurrence of vascular complications, providing that tight glycemic control can be achieved without hypoglycemia or other significant adverse effects. The Japanese Diabetes Society (JDS) recommends glycated hemoglobin (HbA1c) <7.0 % [National Glycohemoglobin Standardization Program (NGSP)] as a general glycemic goal for patients with T2DM. However, many patients in Japan do not achieve this goal [3, 4]. For people with diabetes who do not achieve glycemic control with lifestyle changes including diet and exercise, the JDS recommends treatment with oral antidiabetes drugs (OADs) that should be selected based on the individual patients’ clinical profile. Furthermore, for patients inadequately controlled on monotherapy, the guideline recommends combination therapy with a second drug having a different mode of action [1].

Treatment with dipeptidyl peptidase-4 (DPP-4) inhibitor as monotherapy has been used in Japan since 2009, and recently patients are increasingly being treated with combination of DPP-4 inhibitors and other OADs [3, 5]. DPP-4 inhibitors maintain the concentrations of incretins, glucagon-like peptide-1 (GLP-1) and glucose-dependent insulinotropic polypeptide, especially during the postprandial period. Thus, like GLP-1 receptor agonists, DPP-4 inhibitors reduce fasting and postprandial blood glucose levels through the effect of incretins on increasing the α-and β-cell sensitivity to glucose levels [6, 7]. DPP-4 inhibitors are generally weight-neutral and have a low risk of hypoglycemia. They are not also associated with the adverse gastrointestinal effects reported with GLP-1 receptor agonists [8].

Vildagliptin, a DPP-4 inhibitor, was launched in Japan in 2010. In large global studies with a predominantly Caucasian population, vildagliptin has been demonstrated to be well tolerated and efficacious, as monotherapy and in combination with metformin (Met), sulfonylurea (SU), thiazolidinedione (TZD), or insulin [9–13]. Some reports suggested that Asian patients with T2DM have a more prominent insulin secretory defect than Caucasian patients [14]. Also, Japanese regulatory requirements [15] mandated that the indication for combination therapy with other OADs should be supported with data from the Japanese population; this is to ensure there are no marked differences in the safety and efficacy profiles of OADs drugs among different ethnicities. In a previously published Japanese study, vildagliptin demonstrated stable improvements in HbA1c levels, with relatively low hypoglycemic risk, either as monotherapy or as an add-on to SU for treatment duration of up to 52 weeks [16]. Interestingly, the blood-glucose lowering effect appeared to be numerically better in the Japanese population than in the general global population, which was largely derived from non-Asian populations.

The present study reviewed the results of two open-label studies [16, 17] to provide insights on the long-term efficacy and safety of vildagliptin in combination with other OADs in Japanese patients with T2DM with inadequate glycemic control on OAD monotherapy. These studies have been previously published in Japanese.

## Methods

Two multicenter, open-label studies were included to evaluate the long-term tolerability and safety of vildagliptin as an add-on to other OAD. Study A, in which vildagliptin was added to SU, was completed in 2007 [16], and Study B, in which vildagliptin was added to other OAD [Met, TZD, glinide, or α-glucosidase inhibitor (α-GI)] was completed in 2012 [17]. Inclusion and exclusion criteria were similar in both the studies; patients aged ≥20 years, inadequately controlled [HbA1c (NGSP) ≥6.9 % and ≤10.5 % and fasting plasma glucose (FPG) <270 mg/dL] with OAD monotherapy in addition to diet/exercise therapy were enrolled. The common exclusion criteria of the studies were as follows; pregnant or lactating women, patients with a history of type 1DM or secondary DM, acute metabolic complications within past 24 weeks, acute infection within past 4 weeks, abnormal value in clinical testing (aspartate aminotransferase or alanine aminotransferase activities > 2-2.5 times the upper limit of normal, high level of serum creatinine > 2 mg/dL, or fasting triglyceride > 500–700 mg/dL. After the patient provided written informed consent, vildagliptin [50 mg twice daily (bid)] was administered in addition to OAD for 52 weeks.

For each OAD therapy, changes in HbA1c levels from baseline to 52 weeks or study endpoint were examined. Changes from baseline to endpoint in FPG, fasting insulin, fasting lipids, homeostasis model assessments for β-cell function (HOMA-β) and insulin resistance (HOMA-IR), and body weight were also evaluated. In addition, the proportion of responders, defined as achieving HbA1c <6.9 % at endpoint or a ≥1.0 % and ≥0.5 % reduction in HbA1c from baseline to endpoint were calculated. When parameters were not measured at endpoint, missing values were imputed using the last observation carried forward method. No hypothesis testing was performed and the data were summarized descriptively by treatment. Safety analysis included recording of treatment-emergent adverse events (AEs) and serious AEs (SAEs). Both studies were approved by the institutional review board at each institute which participated in the studies and all the subjects enrolled gave written informed consent prior to start of administration of the study drug. Also, the studies were conducted in accordance with the Helsinki declaration and good clinical practices.

## Results

### Patient characteristics

Baseline characteristics of patients by OAD therapy group are presented in Table 1. Overall, 299 patients were enrolled: 54 were on SU, 58 were on Met, 62 were on TZD, 62 were on α-GI, and 63 were on glinide. The mean age was ~60 years, and the mean body mass index (BMI) was ~25 kg/m^2^. For patients treated with SU (Study A), the mean baseline HbA1c levels and the mean duration of T2DM were numerically higher and longer, respectively, compared with patients in the other OAD groups. As one patient enrolled in the add-on to SU group did not receive vildagliptin, 53 patients were analyzed for safety and efficacy assessments.

**Table 1:** Patient demographics and baseline characteristics

	Study A	Study B			
Parameters	SU	Met	TZD	α-GI	Glinide
	*n* = 54	*n* = 58	*n* = 62	*n* = 62	*n* = 63
Gender, n (%)					
Men	39 (72.2)	35 (60.3)	50 (80.6)	42 (67.7)	45 (71.4)
Women	15 (27.8)	23 (39.7)	12 (19.4)	20 (32.3)	18 (28.6)
Age (years)	60.6 ± 10.24	58.0 ± 11.06	59.0 ± 11.24	60.9 ± 10.41	59.9 ± 12.10
BMI (kg/m^2^)	24.7 ± 3.10	26.0 ± 3.47	26.5 ± 3.77	24.8 ± 3.73	25.0 ± 3.55
Duration of type 2 diabetes mellitus (years)	9.1 ± 6.61	6.8 ± 5.91	6.6 ± 4.90	6.9 ± 5.25	5.7 ± 4.76
HbA1c^a^ (%)	8.0 ± 0.71	7.80 ± 0.87	7.80 ± 0.91	7.66 ± 0.76	7.97 ± 0.89
Fasting plasma glucose (mg/dL)	153.6 ± 28.26	156.1 ± 32.97	155.5 ± 32.16	155.2 ± 28.18	177.7 ± 51.10
GFR (MDRD) category, n (%)					
>80 mL/min/1.73 m^2^	48 (88.9)	52 (89.7)	58 (93.5)	52 (83.9)	53 (84.1)
≤80 mL/min/1.73 m^2^	6 (11.1)	6 (10.3)	4 (6.5)	10 (16.1)	10 (15.9)

Values are expressed as n (%) or mean ± standard deviation

One of 54 patients enrolled in SU add-on therapy did not receive the study drug (vildagliptin); therefore, 53 patients were analyzed for safety and efficacy

^a^HbA1c calculated from JDS value to NGSP value: HbA1c (NGSP) (%) = 1.02 × HbA1c (JDS) (%) + 0.25 %

α-*GI* alpha-glucosidase inhibitor, *BMI* body mass index, *GFR* glomerular filtration rate, *Met* metformin

*MDRD* modification of diet in renal disease, *SU* sulfonylurea, *TZD* thiazolidinedione

### HbA1c

**Fig. 1:**
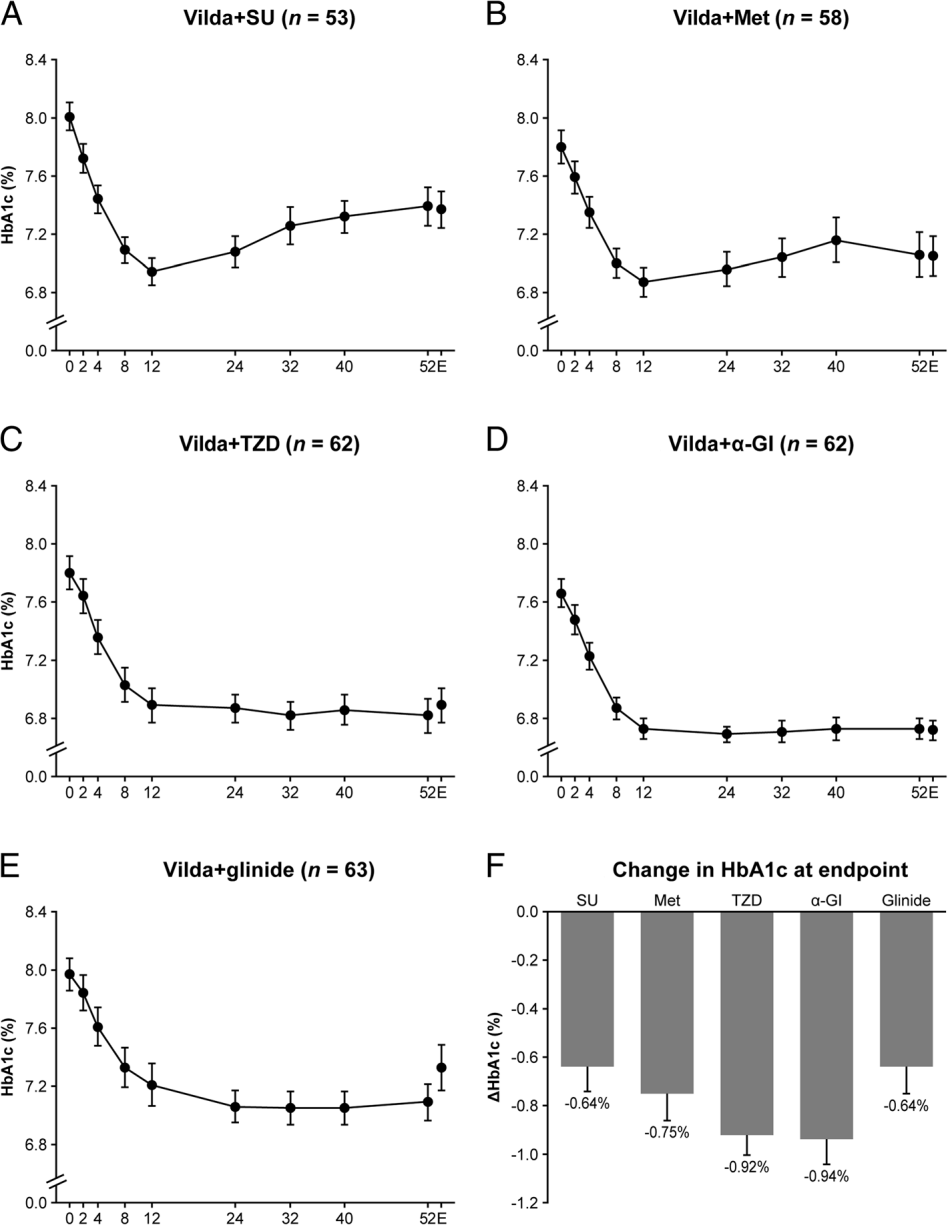
Time-course change in mean glycated hemoglobin (HbA1c) over 52 weeks in patients with type 2 diabetes mellitus treated with vildagliptin as add-on to other oral antidiabetes drugs. **a**: Vildagliptin (Vilda) with sulfonylurea (SU), **b**: Vilda with metformin (Met), **c**: Vilda with thiazolidinedione (TZD), **d**: Vilda with α-glucosidase inhibitor (α-GI), **e**: Vilda with glinide, **f**: ΔHbA1c, change in HbA1c at endpoint; *E* endpoint. Data are presented as mean ± standard error

As shown in Fig. 1, HbA1c decreased rapidly within the initial three months after addition of vildagliptin in all groups, and the time course of change in HbA1c thereafter was similar in all groups. The decrease in HbA1c after 12 weeks of vildagliptin treatment in each group ranged between 0.82 % – 1.09 %. At endpoint, HbA1c reductions in the add-on to SU, Met, TZD, α-GI, and glinide, groups were 0.64 %, 0.75 %, 0.92 %, 0.94 %, and 0.64 %, respectively.

### Responders

The percentage of responders after add-on treatment with vildagliptin is shown in Table 2. The proportion of patients achieving HbA1c <6.9 % (≤6.9 % for add-on to SU) was relatively low in the add-on to SU group (34.6 %) and the add-on to glinide group (37.9 %), but was >50 % in the other OAD groups. The proportion of patients achieving an HbA1c reduction of ≥1.0 % was also low in the add-on to SU group (26.4 %); whereas the proportion in other groups, including the add-on to glinide group was ~40 %.

**Table 2:** Proportion of responders at endpoint

	Study A	Study B			
	SU	Met	TZD	α-GI	Glinide
	*n* ^a^ = 53	*n* ^a^ = 58	*n* ^a^ = 62	*n* ^a^ = 62	*n* ^a^ = 63
	% (*n*)	% (*n*)	% (*n*)	% (*n*)	% (*n*)
HbA1c <6.9 % or ≤6.9%^b^	34.6	53.8	59.3	69.6	37.9
	(18/52)	(28/52)	(32/54)	(39/56)	(22/58)
≥1.0 % reduction in HbA1c	26.4	46.6	41.9	43.5	38.1
	(14/53)	(27/58)	(26/62)	(27/62)	(24/63)
≥0.5 % reduction in HbA1c	60.4	69.0	74.2	80.6	61.9
	(32/53)	(40/58)	(46/62)	(50/62)	(39/63)

^a^Number of patients with both baseline and endpoint HbA1c measurements in the specified population, which was used as denominator, unless specified otherwise

^b^Denominator consists of patients with baseline HbA1c ≥6.9 % and endpoint HbA1c measurement. In SU group, HbA1c ≤6.9 % was employed as criteria of responder

α-*GI* alpha-glucosidase inhibitor, *Met* metformin, *SU* sulfonylurea, *TZD* thiazolidinedione

### Body weight

The mean body weight at endpoint was increased slightly in all the groups, however an increase of more than 2 kg was observed only in the add-on to TZD group (Table 3).

**Table 3:** Change in body weight

	Study A	Study B			
	SU	Met	TZD	α-GI	Glinide
	*n* = 53	*n* = 58	*n* = 62	*n* = 62	*n* = 63
Baseline (kg)	66.01 ± 1.65	68.76 ± 1.285	72.29 ± 1.749	65.60 ± 1.634	66.81 ± 1.728
Change at endpoint (kg)	1.49 ± 0.26	0.51 ± 0.330	2.11 ± 0.318	0.45 ± 0.323	1.17 ± 0.281

Values are expressed mean ± standard error

α-*GI* alpha-glucosidase inhibitor, *Met* metformin, *SU* sulfonylurea, *TZD* thiazolidinedione

### FPG, Fasting Insulin, HOMA-β, HOMA-IR and Fasting Lipids

After vildagliptin co-administration, mean FPG decreased in all the add-on groups. The baseline FPG level was higher in the add-on to glinide group than in the other OAD groups, and the change from baseline to endpoint was relatively small in the add-on to SU group (Table 4). Fasting insulin levels increased in the add-on to SU group and slightly increased or decreased in other OAD groups. HOMA-β increased in all the OAD groups, and the change was greatest in the add-on to SU group. The value change in HOMA-IR increased only in the add-on to SU group and decreased in all the other OAD groups.

**Table 4:** Change in fasting plasma glucose, fasting insulin, HOMA-β and HOMA − IR

	Study A	Study B			
	SU	Met	TZD	α-GI	Glinide
	*n* = 53	*n* = 58	*n* = 62	*n* = 62	*n* = 63
Fasting plasma glucose (mg/dL)					
Baseline	153.0 ± 3.88	156.1 ± 4.33	155.5 ± 4.08	155.2 ± 3.58	177.7 ± 6.44
Δ change	−6.6 ± 3.76	−14.0 ± 3.78	−19.6 ± 2.94	−17.0 ± 3.49	−18.8 ± 4.60
Fasting insulin (μU/L)					
Baseline	7.90 ± 0.56	8.33 ± 0.616	6.43 ± 0.536	7.58 ± 0.691	7.13 ± 0.638
Δ change	1.92 ± 0.93	−0.23 ± 0.478	−0.56 ± 0.298	0.02 ± 0.383	−0.04 ± 0.459
HOMA-β					
Baseline	33.10 ± 2.41	35.09 ± 3.28	28.13 ± 2.67	32.83 ± 4.08	25.66 ± 2.39
Δ change	11.30 ± 2.89	6.76 ± 3.34	5.79 ± 1.94	4.64 ± 2.06	4.50 ± 1.61
HOMA-IR					
Baseline	3.07 ± 0.26	3.31 ± 0.29	2.42 ± 0.20	2.89 ± 0.25	3.26 ± 0.39
Δ change	0.76 ± 0.56	−0.36 ± 0.19	−0.53 ± 0.12	−0.23 ± 0.17	−0.43 ± 0.26

Values are expressed as mean ± standard error

Δ change from baseline to endpoint, α-*GI* alpha-glucosidase inhibitor, *HOMA* homeostasis model assessment, *Met* metformin, *SU* sulfonylurea, *TZD* thiazolidinedione

As shown in Table 5, triglyceride, and total cholesterol levels decreased from baseline to endpoint in all OAD groups. Low-density lipoprotein cholesterol levels increased in the add-on to SU group and decreased in the other OAD groups. High-density lipoprotein cholesterol levels slightly increased in the add-on to α-GI group and decreased in the other OAD groups.

**Table 5:** Change in fasting lipids

	Study A	Study B			
	SU	Met	TZD	α-GI	Glinide
	*n* = 53	*n* = 58	*n* = 62	*n* = 61^a^	*n* = 61^a^
Triglyceride (mg/dL)					
Baseline	137.9 ± 13.09	158.0 ± 12.48	131.6 ± 11.69	150.5 ± 17.80	155.8 ± 13.68
Δ change	−6.6 ± 9.28	−19.4 ± 11.42	−7.8 ± 7.93	−12.3 ± 12.90	−8.7 ± 10.21
Total cholesterol (mg/dL)					
Baseline	197.8 ± 3.95	195.0 ± 4.19	199.0 ± 4.45	200.0 ± 4.47	206.9 ± 4.98
Δ change	−3.3 ± 3.22	−7.1 ± 2.40	−7.2 ± 3.74	−5.3 ± 3.53	−7.6 ± 3.37
Low-density lipoprotein (mg/dL)					
Baseline	123.8 ± 3.81	115.8 ± 3.51	115.3 ± 3.60	122.7 ± 4.22	126.1 ± 4.21
Δ change	1.3 ± 2.77	−0.9 ± 2.44	−1.5 ± 3.19	−1.2 ± 3.31	−2.3 ± 3.10
High-density lipoprotein (mg/dL)					
Baseline	51.5 ± 1.45	55.4 ± 1.85	62.5 ± 2.54	54.1 ± 1.58	57.2 ± 1.76
Δ change	−1.8 ± 0.74	−1.4 ± 1.03	−2.0 ± 1.22	0.4 ± 0.82	−1.1 ± 1.21

Values are expressed as mean ± standard error

^a^Measurements for one patient in the α-GI group and two patients in the glinide group were missing

Δ change from baseline to endpoint, α-*GI* alpha-glucosidase inhibitor, *Met* metformin, *SU* sulfonylurea, *TZD* thiazolidinedione

### Adverse events

The incidence of AEs was comparable among the OAD groups: 90.6 % in the add-on to SU group, 94.8 % in the add-on to Met group, 83.9 % in the add-on to TZD group, 85.5 % in the add-on to α-GI group, and 82.5 % in the add-on to glinide group (Table 6). The most frequent AE was nasopharyngitis in all the OAD groups. The majority of AEs were mild or moderate in severity and no death was reported during the studies. The incidence of adverse drug reactions was 47.2 % in the add-on to SU group, 29.3 % in the add-on to Met group, 24.2 % in the add-on to TZD group, 12.9 % in the add-on to α-GI group and 15.9 % in the add-on to glinide group. At least one episode of hypoglycemic symptoms was reported in 2 patients (3.8 %) in the add-on to SU group and 1 patient (1.7 %) in the add-on to Met group. The hypoglycemic events were moderate in severity and categorized as grade 1. None of the patients in the TZD, α-GI, or glinide groups reported hypoglycemic events. Discontinuation of treatment due to AEs was overall low, occurred in 4 patients (7.5 %) in the add-on to SU group, 3 patients (5.2 %) in the add-on to Met group, 4 patients (6.5 %) in the add-on to TZD group, 4 patients (6.5 %) in the add-on to α-GI group, and 6 patients (9.5 %) in the add-on to glinide group.

**Table 6:** Adverse events

	Study A	Study B			
*n* (%)	SU	Met	TZD	α − GI	Glinide
	*n* = 53	*n* = 58	*n* = 62	*n* = 62	*n* = 63
Adverse events (AEs)	48 (90.6)	55 (94.8)	52 (83.9)	53 (85.5)	52 (82.5)
Adverse drug reactions	25 (47.2)	17 (29.3)	15 (24.2)	8 (12.9)	10 (15.9)
Serious AEs	3 (5.7)	4 (6.9)	5 (8.1)	4 (6.5)	2 (3.2)
Discontinuation due to AEs	4 (7.5)	3 (5.2)	4 (6.5)	4 (6.5)	6 (9.5)
Patients with at least one episode of hypoglycemic symptoms	2 (3.8)	1 (1.7)	0 (0.0)	0 (0.0)	0 (0.0)
AEs by preferred term >5 %					
Nasopharyngitis	25 (47.2)	17 (29.3)	13 (21.0)	25 (40.3)	20 (31.7)
Constipation	4 (7.5)	9 (15.5)	3 (4.8)	4 (6.5)	5 (7.9)
Back pain	8 (15.1)	3 (5.2)	4 (6.5)	5 (8.1)	3 (4.8)
Gastritis	6 (11.3)	5 (8.6)	4 (6.5)	2 (3.2)	1 (1.6)
Upper respiratory tract infection	1 (1.9)	3 (5.2)	4 (6.5)	1 (1.6)	4 (6.3)
Dizziness	5 (9.4)	2 (3.4)	2 (3.2)	2 (3.2)	5 (7.9)
Contusion	1 (1.9)	3 (5.2)	1 (1.6)	1 (1.6)	5 (7.9)
Bronchitis	1 (1.9)	5 (8.6)	0 (0.0)	3 (4.8)	1 (1.6)
Edema peripheral	0 (0.0)	1 (1.7)	4 (6.5)	2 (3.2)	1 (1.6)
Blood amylase increased	3 (5.7)	3 (5.2)	3 (4.8)	2 (3.2)	0 (0.0)
Osteoarthritis	1 (1.9)	3 (5.2)	3 (4.8)	0 (0.0)	2 (3.2)
Headache	1 (1.9)	3 (5.2)	1 (1.6)	1 (1.6)	3 (4.8)
Diarrhea	0 (0.0)	5 (8.6)	1 (1.6)	1 (1.6)	0 (0.0)
Hunger	7 (13.2)	3 (5.2)	1 (1.6)	1 (1.6)	2 (3.2)
Hypoesthesia	1 (1.9)	0 (0.0)	3 (4.8)	0 (0.0)	4 (6.3)
Conjunctivitis allergic	0 (0.0)	4 (6.9)	0 (0.0)	0 (0.0)	2 (3.2)
Periodontitis	0 (0.0)	0 (0.0)	1 (1.6)	4 (6.5)	1 (1.6)
Dry eye	0 (0.0)	0 (0.0)	4 (6.5)	0 (0.0)	1 (1.6)
C-reactive protein increased	2 (3.8)	4 (6.9)	0 (0.0)	0 (0.0)	1 (1.6)
Arthralgia	3 (5.7)	3 (5.2)	1 (1.6)	0 (0.0)	1 (1.6)
Pharyngitis	1 (1.9)	10 (17.2)	3 (4.8)	1 (1.6)	1 (1.6)
Blood creatine phosphokinase increased	7 (13.2)	2 (3.4)	2 (3.2)	1 (1.6)	1 (1.6)
Tremor	7 (13.2)	1 (1.7)	1 (1.6)	0 (0.0)	0 (0.0)
Asthenia	6 (11.3)	2 (3.4)	0 (0.0)	2 (3.2)	2 (3.2)
Blood creatine phosphokinase MB increased	5 (9.4)	2 (3.4)	1 (1.6)	1 (1.6)	0 (0.0)
Hyperhidrosis	4 (7.5)	2 (3.4)	0 (0.0)	0 (0.0)	2 (3.2)
Hypertension	3 (5.7)	2 (3.4)	2 (3.2)	2 (3.2)	2 (3.2)
Eczema	3 (5.7)	2 (3.4)	2 (3.2)	2 (3.2)	2 (3.2)
Myalgia	3 (5.7)	1 (1.7)	1 (1.6)	0 (0.0)	1 (1.6)
Palpitations	3 (5.7)	1 (1.7)	1 (1.6)	3 (4.8)	1 (1.6)
Anemia	3 (5.7)	1 (1.7)	0 (0.0)	2 (3.2)	0 (0.0)

α-*GI* alpha-glucosidase inhibitor, *Met* metformin, *SU* sulfonylurea, *TZD* thiazolidinedione

## Discussion

The efficacy and safety analysis from two long-term 52-week studies showed that vildagliptin 50 mg bid, in combination with other OADs in Japanese patients with T2DM, exerts robust blood glucose-lowering effects and is well tolerated. There was no remarkable difference in the incidence of AEs among the OADs used as baseline therapy. The risk of hypoglycemia was overall low, with a slight increase in the add-on to SU group. The events were mild in severity and were manageable by the patients.

DPP-4 inhibitors are relatively new drugs among OADs, however, their use in T2DM patients is markedly increasing in the clinical setting. The blood-glucose lowering effect of DPP-4 inhibitor has been well recognized, but only few studies to investigate the differences among DPP-4 inhibitors are available [18]. An indirect comparison adjusted for the background characteristics in Japanese patients with T2DM revealed that the effect of vildagliptin (50 mg bid) in reducing HbA1c levels was significantly stronger compared to sitagliptin (50 or 100 mg qd) [19]. Concerning the mode of enzyme inhibition by DPP-4 inhibitors, the inhibition kinetics of vildagliptin was slower than that of sitagliptin [20, 21]. This difference in the kinetics of DPP-4 inhibition may be related with the significant suppression of blood glucose fluctuations during 24 h with vildagliptin compared to sitagliptin [22]. However, HbA1c prior to treatment with DPP-4 inhibitors is strongly associated with the variance of HbA1c reduction in response to DPP-4 inhibitors [23]. The efficacy and safety of vildagliptin in long-term observation has remained to be elucidated thoroughly.

Addition of vildagliptin 50 mg bid resulted in a rapid decrease in HbA1c in all the OAD groups. The combination of vildagliptin and other OADs provided an effective glucose-lowering therapy, but attention should be paid to hypoglycemic events as well as refractoriness in the reduction of HbA1c levels, especially when vildagliptin is administered in combination with insulin secretagogues for an extended period. In the current studies, the degree of HbA1c reduction by vildagliptin in combination with insulin secretagogues (SU or glinide) was relatively smaller when compared to the combination with non-insulin secretagogues (Met, TZD, or α-GI).

In addition to hypoglycemia, weight gain is another important issue to be considered while choosing pharmacotherapy for the management of T2DM [24]. In these studies, a >1 kg increase in weight was found with the add-on to insulin secretagogues (SU, glinide) and TZD therapy, in contrast to a previous study on sitagliptin, where weight reduction was observed due to a decrease in dose of SU [25]. The increase in body weight in the add-on to insulin secretagogues group is presumably due to defensive eating secondary to the increased tendency to mild hypoglycemia in the SU and glinide groups; in the TZD group it is not clear why the usual increase in weight is exacerbated by the addition of vildagliptin when one considers its mechanisms to mitigate body weight [26].

One limitation of this manuscript is that two independent clinical studies were reviewed in a parallel manner; hence, no statistical analysis was performed to compare efficacy and safety parameters among the different treatment groups. Another limitation is that only combination of vildagliptin with OAD was focused in this article, although it has been reported that vildagliptin as add-on to insulin significantly reduced HbA1c in Japanese patients with T2DM [27]. Concerning the safety of DPP-4 inhibitors, no studies have revealed that DPP-4 inhibitors provide beneficial outcome on incidence of cardiovascular events, however, meta-analysis have shown that DPP-4 inhibitors have a neutral effect on major cardiovascular events [28, 29].

## Conclusions

In conclusion, vildagliptin as an add-on to other OADs in Japanese patients with T2DM results in robust decrease in HbA1c levels with good tolerability and low risk of hypoglycemia and weight gain. Vildagliptin improved glucose metabolism regardless of the type of OADs combined with vildagliptin. Vildagliptin is considered to be a clinically reasonable treatment option with good tolerability profile for patients with T2DM responding inadequately to other OADs.

## Abbreviations

AEs, Adverse events; BMI, Body mass index; DPP-4, Dipeptidyl peptidase-4; E, Endpoint; GFR, Glomerular filtration rate; GLP-1, Glucagon-like peptide-1; HbA1c, Glycated hemoglobin; HOMA-IR, Homeostasis model assessments for insulin resistance; HOMA-β, Homeostasis model assessments for β-cell function; JDS, Japanese Diabetes Society; MDRD, modification of diet in renal disease; Met, Metformin; NGSP, National Glycohemoglobin Standardization Program; OADs, Oral antidiabetes drugs; SAEs, Serious adverse events; SU, Sulfonylurea; T2DM, Type 2 diabetes mellitus; TZD, Thiazolidinedione; α-GI, α-glucosidase inhibitor
